# Spontaneous diuresis in combination with furosemide stress test (SD-FST) as predictor for successful liberation from kidney replacement therapy: a prospective observational study

**DOI:** 10.1186/s13054-025-05452-1

**Published:** 2025-05-26

**Authors:** Lorenz Weidhase, Stephanie Wille, Helene Foede, Fanny Gilch, Meinhard Mende, Christina Scharf-Janßen, Sirak Petros, Jonathan de Fallois

**Affiliations:** 1 Medical Intensive Care Unit, University Hospital Leipzig, Leipzig, Germany; 2 Institute for Medical Informatics, Statistics and Epidemiology, University Leipzig, Leipzig, Germany; 3 Department of Anesthesiology, University Hospital, LMU Munich, Munich, Germany; 4 Medical Department III, Division of Nephrology, University Hospital Leipzig, Leipzig, Germany

**Keywords:** Furosemide stress test (FST), Kidney replacement therapy (KRT), Acute kidney injury (AKI), Recovery of kidney function

## Abstract

**Background:**

The optimal time for initiating kidney replacement therapy (KRT) in acute kidney injury (AKI) has been extensively studied in recent years. In contrast, there are currently insufficient data on the best time to discontinue KRT. One diagnostic option to unmask tubular reserve and indirectly estimate the glomerular filtration rate is the furosemide stress test (FST).

**Methods:**

We conducted a prospective, observational single-center trial. A FST was carried out in patients who developed spontaneous diuresis (SD) during ongoing KRT with a urine output of at least 400 ml in 24 h without any diuretic therapy. A positive FST was defined with urine output > 200 ml within 2 h following intravenous furosemide application. Follow-up was performed for 7 days and the need to restart KRT was assessed daily.

**Results:**

After 100 patients were enrolled in the trial, 98 patients were eligible for further evaluation. 76 patients were FST-positive, while 22 patients were FST-negative. Resumption of KRT within the 7-day follow-up was required in only 14.5% of the FST-positive, but 72.7% of the FST-negative patients (*p *< 0.001). The urine output after FST was also significantly associated with successful release from KRT (AUC 0.87; *p* < 0.001).

**Conclusions:**

In critically ill patients with recovery of SD > 400ml/d during ongoing KRT, the FST helps to identify patients who can be successfully liberated from KRT. By detecting the tubular reserve using FST, the possibility of short-term kidney recovery after AKI can be estimated.

**Trial registration:**

German Clinical Trials Registry (DRKS00030560); date of registration 18/11/2022. https://drks.de/search/de/trial/DRKS00030560.

**Supplementary Information:**

The online version contains supplementary material available at 10.1186/s13054-025-05452-1.

## Background

The management of acute kidney injury (AKI) remains a challenge in critical care medicine. AKI is common in critically ill and is associated with high mortality, which increases particularly when kidney replacement therapy (KRT) is required [1–4].

The optimal time to initiate KRT has been extensively studied in recent years. Delaying KRT may be justified in certain cases, because renal function may recover spontaneously [5–9]. However, KRT should not be postponed if mandatory indications are observed [10].

In contrast, there are currently insufficient data on the best time to discontinue KRT. This issue is highly relevant, since shorter dialysis time is cost-effective and possible complications of the procedure could be avoided [6, 9, 11, 12]. Patient factors that can be associated with non-recovery after AKI include for example age, chronic kidney disease, or surgical versus non-surgical admission [13, 14]. Furthermore, the severity of the acute illness [15], high urine output [15–19], KRT duration [19], decreasing serum creatinine [15, 16, 19, 20], urinary creatinine and urea [18, 21], kinetic of estimated glomerular filtration rate (eGFR) [17] or biomarker-based estimates may be helpful in decision-making to discontinue KRT [20, 22–24].

A large multicenter observational study found a 24-h spontaneous diuresis (SD) of at least 436 ml without diuretics to be the most important predictor for successful discontinuation of KRT [25], similar findings were found for increased urine output with diuretics [26].

Therefore, this could be used as a diagnostic test, although this association was not consistently demonstrated [27]. A diagnostic candidate for unmasking the tubular reserve and thus indirectly estimating the glomerular filtration rate (GFR) is the furosemide stress test (FST). The test has been shown to help to identify patients with severe and progressive AKI and improve the timing for KRT initiation [24, 28–31]. Current data suggest that FST may also be a promising candidate for predicting successful recovery of renal function after KRT [32].

Given this background, we hypothesized that a combination of re-onset SD and FST can predict renal function recovery after KRT with a high degree of accuracy. This may help to develop reliable predictors for liberation from KRT.

## Methods

### Study design

This study is a prospective, observational single-center pilot trial registered at the German trial registry (DRKS00030560) and approved by the Institutional Review Board (IRB) at the University of Leipzig, Germany (171/22-ek). The study was conducted according to guidelines for reporting observational studies (STROBE) (Additional file 1).

We planned to enroll 100 critically ill adult patients treated in the medical intensive care unit (ICU) of the University Hospital Leipzig. A written informed consent was obtained from all patients or their legal guardians. Patients were recruited sequentially and subsequently followed up prospectively. Since knowledge of the exact fluid balance by the treatment team is essential in critically ill patients, blinding was not possible. Enrollment of participants and the implementation of the intervention were carried out by trained medical staff. The study received no funding.

### Patients

All critically ill patients with severe AKI and requirement for KRT were screened. Patients were included in the study when SD resumed during ongoing KRT with a urine volume of at least 400 ml in 24 h without any diuretic therapy. Exclusion criteria were refusal of study participation, age < 18 years, pregnancy and lactation, and preexisting residual kidney function (eGFR < 15 ml/min/1.73 m^2^).

### Data collection

Demographic and clinical data were collected at the time of ICU admission. These included age, gender, height, weight, body mass index (BMI), Acute Physiology And Chronic Health Evaluation (APACHE)-II score [33], Sequential Organ Failure Assessment (SOFA) score [34], mean arterial blood pressure (MAP), serum creatinine, AKI stage according KDIGO guidelines, need for mechanical ventilation, need for vasopressors, diagnosis of sepsis according to the sepsis-3 criteria [35], cardiopulmonary resuscitation, transfusion-requiring bleeding on the day of admission, fluid balance within the first 24 h, and preexisting comorbidities.

At the beginning of KRT, serum creatinine, urea, pH, potassium, bicarbonate, 24-h urine output, fluid balance, and presence of hypervolemia were documented. Clinical variables and SD of the last 24 h before the FST, as well as the administered furosemide dose and urine output thereafter were also recorded.

Outcome and safety parameters were taken from electronic patient records or generated through telephone calls to patients, legal representatives or managing physicians.

### Procedure

The KRT was administered as continuous (CVVHD-CiCa®) or intermittent (Genius®, both: Fresenius medical care, Bad Homburg, Germany) dialysis depending on the patient's condition. Some patients were first treated continuously and then intermittently. The dialysis dose was usually set at 25 ml/kg/h for CVVHD, and at 90 l in 8–12 h every other day for intermittent dialysis (slow-extended daily dialysis (SLEDD)).

KRT was stopped if the urine output of the previous day was at least 400 ml without any diuretic administration. Furosemide was then administered immediately after collecting a urine sample and emptying the urine bag.

The dose of Furosemide was calculated at 1.5 ml/kg ideal body weight or, in obese patients, adjusted body weight [28]. We applied the Hamwi equation to calculate the ideal body weight (for males: 48 kg for the first 152 cm + 1.1 kg for each additional cm; for females 45 kg for the first 152 cm + 0.9 kg for each additional cm) [36, 37]. After 2 h, the urine volume was measured.

The FST was considered positive if the urine output was at least 200 ml or more in the first 2 h after furosemide administration.

The absence of KRT restart within 7 days following FST was evaluated daily. Urine output, serum potassium, pH, bicarbonate, creatinine, urea, fluid balance, and symptoms of uremia were also recorded daily. The decision to restart KRT was made by an independent intensive care physician.

Finally, ICU, hospital, 28-day and 90-day survival as well as requirement for KRT on day 90 were recorded.

### Endpoints

We hypothesized that combining a diagnostic test with the sign of spontaneous renal recovery could improve the diagnostic yield to uncover the success of liberation from KRT. Therefore, the primary endpoint was defined as successful liberation from KRT within 7 days after recovery of SD and FST. As a secondary endpoint we investigated if other parameters could also increase the predictive probability of successful liberation from KRT. For this purpose, the parameters collected at resumption of SD before FST were analyzed.

Safety endpoints included survival data (ICU, hospital, 28 d, 90 d) and requirement of KRT until day 90.

### Laboratory analyses

All biochemical analyses were performed at the Institute of Laboratory Medicine, Clinical Chemistry and Molecular Diagnostics, Leipzig University Hospital. Serum samples were analyzed shortly after blood withdrawal on a Cobas 8000 automated laboratory analyzer (Roche Diagnostics, Mannheim, Germany) in accordance with the manufacturer's protocol.

### Adverse events of FST

Possible side effects of furosemide administration were monitored. We compared the occurrence of hypokalemia < 3.5 mmol/l, cardiac arrhythmias, and hypotension with a mean arterial pressure < 65 mmHg as well as the need for potassium supplementation and the maximum noradrenaline dose during the periods 24 h before and 24 h after furosemide administration.

### Statistical analyses

This study was designed as an exploratory trial, as a sample size calculation was not possible due to lack of appropriate published data. Therefore, the aims of the study were primarily exploratory.

Categorical variables were displayed as frequencies with percentages and tested with the Chi-square test (two-sided) or Fisher exact test as appropriate. All continuous variables were tested for normal distribution using the Shapiro–Wilk test. If normal distribution was present, the data was presented as mean and standard deviation and the group comparison was performed using student´s t-test. If there was no normal distribution, the data representation was as median with 25 th and 75 th quantiles in square brackets and group comparisons were performed with the Mann–Whitney U test. To estimate the predictive quality of the FST for a successful liberation from KRT, the sensitivity, specificity as well as the positive and negative predictive values were calculated. The relationship between urine volume after furosemide administration, SD of the previous day, and successful liberation from KRT was investigated using Receiver Operating Characteristic (ROC) analysis and the area under curve (AUC) was calculated.

A logistic regression was applied to test the impact of different parameters at time of FST regarding successful liberation from KRT. Parameters with a significant influence on the need for renewed dialysis on the univariate analysis were integrated into this model after testing multicollinearity. To avoid overfitting a backward selection was performed in the first step, afterwards the significant variables were included to find the most parsimonious model.

The before and after comparisons to quantify possible adverse events caused by Furosemide administration were performed using the McNemar test for dichotomous variables and the Wilcoxon signed-rank test for continuous variables. All analyses were performed using IBM SPSS, version 29 (Minneapolis, USA) and GraphPad Prism, version 10, was applied for the generation of graphs. The significance level was set at *p* < 0.05 for two-sided tests.

## Results

### Patients

**Fig. 1:**
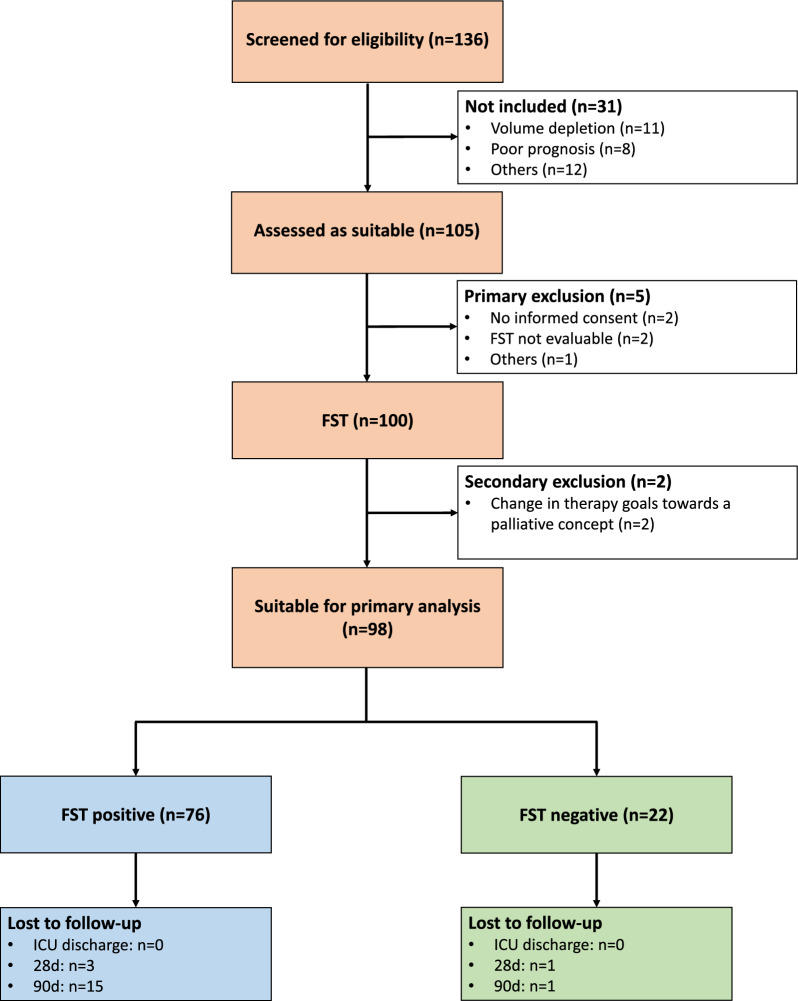
Recruitment flowchart of patients with recovery spontaneous diuresis during ongoing kidney replacement therapy with a urine output of at least 400 ml in 24 h. *FST* Furosemide stress test*, ICU* Intensive care unit

Between November 2022 and September 2024 136 patients were screened. Of these, 31 were primary excluded due to medical reasons. Of the remaining 105 patients five were secondary excluded from study participation. 100 patients received the FST. Finally, 98 patients were eligible for further evaluation, since the treatment goal was changed to a palliative concept in two patients during the follow-up of 7 days after FST (Fig. 1). Of the 98 patients immediately prior to FST, 83/98 had continuous KRT and 15/98 intermittent KRT.

Two hours after the furosemide test dose 76 patients showed a positive FST with a urine output of at least 200 ml. The remaining 22 patients with urine output < 200 ml after 2 h were considered FST negative (Fig. 1). Neither the baseline clinical characteristics and comorbidities (Table 1) nor the reasons to initiate KRT differed between the two groups (Additional file 2). Indications to restart KRT after FST were mainly azotemia, oligo-/anuria, hypervolemia, and encephalopathy (Additional file 3).

**Table 1:** Baseline characteristics of patients developing spontaneous diuresis under ongoing kidney replacement therapy at ICU admission

Parameter	All n = 98	FST positive n = 76	FST negative n = 22	*p*
*General characteristics:*				
Age (years)	64 ± 13	65 ± 13	59 ± 13	0.072
Sex (male) n (%)	69 (70.4)	53 (69.7)	16 (72.7)	0.787
Height (cm)	174 ± 9	173 ± 9	175 ± 8	0.317
Weight (kg)	84 [71; 104]	82 [71; 109]	87 [69; 101]	0.902
BMI (kg/m^2^)	27.9 [23.6; 34.5]	27.9 [23.8; 34.7]	28.0 [22.0; 34.2]	0.574
APACHE II	31 ± 7	31 ± 7	30 ± 9	0.494
SOFA	10 ± 4	10 ± 4	11 ± 4	0.435
MAP (mmHg)	59 [53; 66]	59 [51; 66]	59 [54; 75]	0.682
Creatinine (µmol/l)	208 [133; 375]	193 [126; 355]	273 [136; 497]	0.115
AKI stage 1 n (%)	7 (7.1)	5 (6.6)	2 (9.1)	0.720
AKI stage 2 n (%)	24 (24.5)	20 (26.3)	4 (18.2)	
AKI stage 3 n (%)	67 (68.4)	51 (67.1)	16 (72.7)	
Mechanical ventilation n (%)	36 (36.7)	30 (39.5)	6 (23.7)	0.296
Vasopressors n (%)	59 (60.2)	48 (63.2)	11 (50.0)	0.267
Sepsis n (%)	66 (67.3)	52 (68.4)	14 (63.6)	0.673
CPR n (%)	4 (4.1)	4 (5.3)	0 (0.0)	0.572
Bleeding^#^ n (%)	16 (16.3)	12 (15.8)	4 (18.2)	0.752
Fluid balance*	2133 [391; 4250]	1790 [143; 4243]	2756 [803; 5508]	0.155
*Preexisting conditions:*				
Chronic heart failure (NYHA IV) n (%)	29 (29.6)	23 (30.3)	6 (27.3)	0.787
Pre-existing immunosuppression n (%)	14 (14.3)	11 (14.5)	3 (13.6)	1.000
Liver cirrhosis n (%)	22 (22.4)	17 (22.4)	5 (22.7)	1.000
Active malignancy n (%)	19 (19.4)	17 (22.4)	2 (9.1)	0.227
Diabetes mellitus n (%)	44 (44.9)	35 (46.1)	9 (40.9)	0.669
Hypertension n (%)	62 (63.3)	50 (65.8)	12 (54.5)	0.335

Data presented as *n* (%), mean ± standard deviation or median [25 th, 75 th quantile]

^#^bleeding requiring transfusion; *fluid balance within the first 24 h after admission on ICU; *AKI* Acute kidney injury, *APACHE II* Acute Physiology And Chronic Health Evaluation II, *BMI* Body mass index, *CPR* Cardiopulmonary resuscitation, *eGFR* Estimated glomerular filtration rate, *FST* Furosemide stress test*, MAP* Mean arterial pressure, *NYHA* New York Heart Association, *SOFA* Sequential organ failure assessment

The FST positive patients showed a significant lower SOFA score, higher MAP and lower creatinine level. No differences were found regarding the duration, type and settings of previous KRT. The 24 h-SD before the FST and the applicated furosemide dose were similar between the two groups. As expected, the urine output after the FST was significantly higher in positive tested patients (Table 2).

**Table 2:** Patient characteristics at the time of furosemide stress test and aspects of furosemide stress test

Parameter	All n = 98	FST positive n = 76	FST negative n = 22	*p*
*Pat. characteristics at the time of FST:*				
Mechanical ventilation n (%)	34 (34.7)	23 (30.3)	11 (50.0)	0.087
Vasopressors n (%)	18 (18.4)	13 (17.1)	5 (22.7)	0.543
SOFA	7 [5; 9]	6 [5; 9]	8 [6; 12]	**0.038**
APACHE II	24 ± 6	24 ± 5	24 ± 7	0.913
MAP (mmHg)	81 [71; 92]	84 [72; 96]	73 [63; 80]	**0.004**
Creatinine (µmol/l)	118 [82; 201]	107 [73; 167]	188 [113; 334]	**0.002**
Urea (mmol/l)	11.1 [6.9; 16.6]	11.0 [6.7; 16.1]	11.7 [7.5; 17.5]	0.595
Potassium (mmol/l)	4.3 [4.0; 4.5]	4.2 ± 0.4	4.5 ± 0.6	0.090
pH	7.43 [7.39; 7.47]	7.44 [7.39; 7.47]	7.41 [7.37; 7.46]	0.334
Bicarbonate (mmol/l)	26.8 [24.9; 28.6]	27.1 [25.0; 28.7]	26.0 [23.5; 28.6]	0.124
*KRT before FST*:				
Duration of KRT (d)	4 [3; 6]	4 [3; 6]	4 [2; 9]	0.620
CVVHD-CiCa®	83 (84.7)	68 (89.5)	15 (68.2)	0.075
SLEDD	6 (6.1)	2 (2.6)	4 (18.2)	
CVVHD-CiCa® and SLEDD	9 (9.2)	6 (7.9)	3 (13.6)	
Blood flow rate (ml/min)^#^	100 [100; 120]	100 [100; 115]	100 [100; 133]	0.170
Dialysate flow rate CVVHD-cica® (ml/h)^#^	2000 [2000; 2000]	2000 [2000; 2000]	2000 [2000; 2000]	0.696
Dialysate flow rate SLEDD l/h)^#^	9000 [7875; 9000]	9000 [6000; 9000]	9000 [8250; 10929]	0.432
Ultrafiltration rate (ml/h)^#^	100 [0; 200]	100 [0; 200]	25 [0; 155]	0.054
*Aspects of FST*:				
Dose of furosemide (mg)	120 [100; 130]	120 [100; 134]	110 [100; 123]	0.645
24-h diuresis before FST (ml)	650 [500; 993]	645 [500; 1191]	680 [495; 843]	0.610
2-h diuresis after furosemide (ml)	500 [201; 900]	655 [425; 1000]	93 [50; 120]	** < 0.001**

Significant p-values < 0.05 were highlighted in bold

Data presented as *n* (%), mean ± standard deviation or median [25 th, 75 th quantile]

^#^KRT settings before FST; *FST* furosemide stress test, *SLEDD* slow extended daily dialysis; *APACHE II* Acute Physiology And Chronic Health Evaluation II, *CVVHD-CiCa®* Continuous veno-venous hemodialysis with regional citrate anticoagulation, *FST* Furosemide stress test*, IHD* intermittent hemodialysis*, KRT* Kidney replacement therapy, *MAP* Mean arterial pressure, *SOFA* Sequential organ failure assessment

### FST as a predictor of successful liberation from KRT

**Fig. 2:**
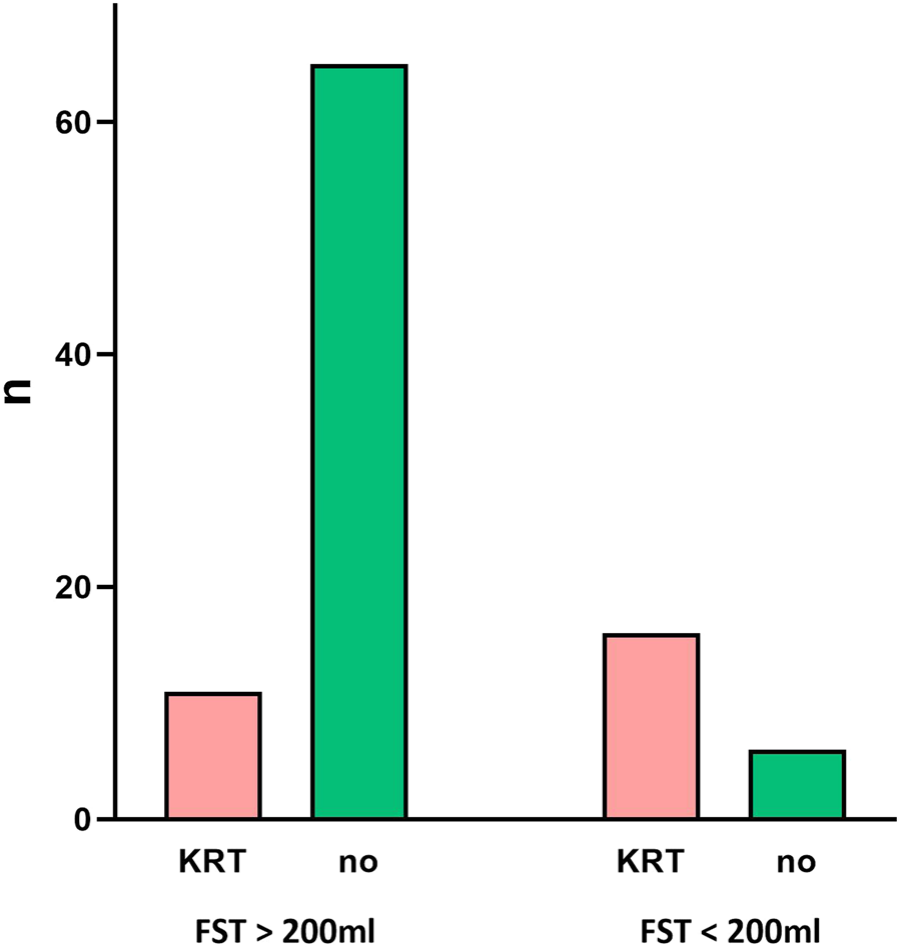
Successful liberation from kidney replacement therapy within 7 days after recovery of spontaneous diuresis and furosemide stress test. Data are presented graphically as a bar chart. All patients (n) had spontaneous diuresis (SD) of at least 400 ml/24 h before the furosemide stress test (FST), *KRT* Kidney replacement therapy

Of the 76 FST positive patients, only 11 required further KRT in the following 7 days (14.5%). In patients with negative FST, there was a remarkable five- to six-fold increase in the risk of requiring restart of KRT (16/22 patients (72.7%)) (Fig. 2). This result was highly significant and differed not in the continuous und intermittent KRT subgroup (*p* < 0.001) (Additional file 4).

A positive FST demonstrated a sensitivity of 0.915 and a specificity of 0.593, with a positive predictive value of 0.855 and a negative predictive value of 0.727 to predict a successful liberation from KRT in the following 7 d.

In comparison, the resumption of SD of at least 400 ml alone showed a lower positive predictive value of 0.724 for a successful liberation from KRT.

**Fig. 3:**
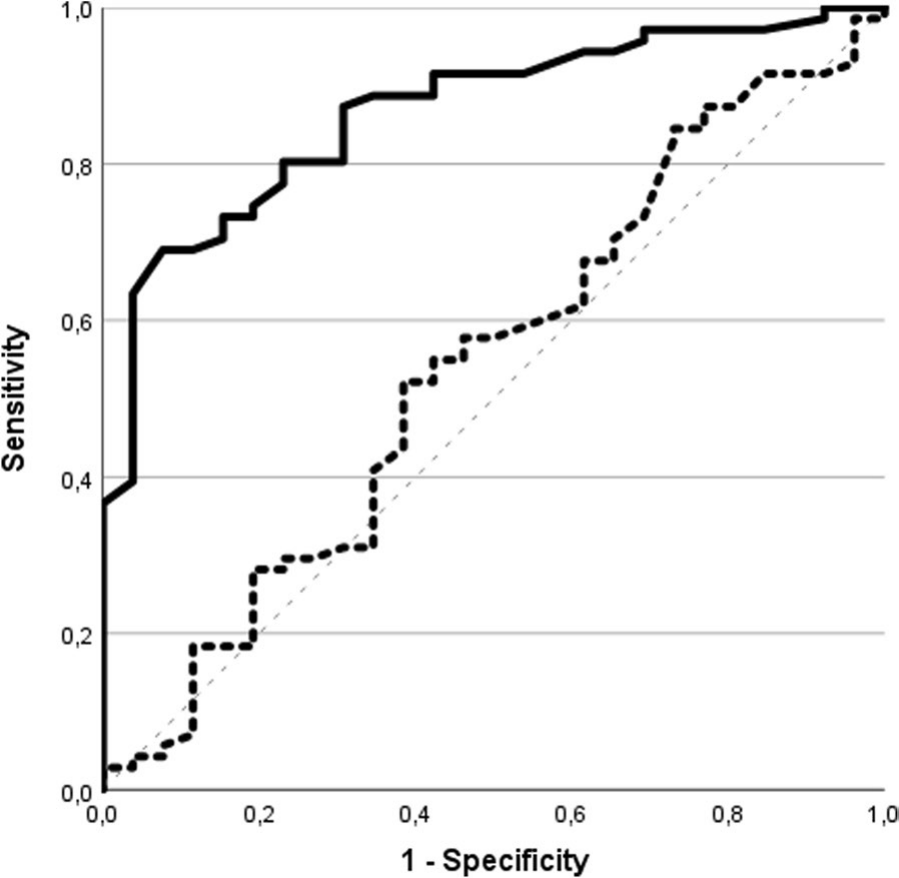
Diuresis and successful liberation from kidney replacement therapy within 7 days. Data are presented graphically as ROC curve. Thick continuous line: Diuresis 2 h after FST and successful liberation from KRT within 7 d (AUC: 0.871, CI 0.799–0.943). Thick dashed line: SD of the previous day (24 h) and successful weaning from KRT within 7 d (AUC 0.542; CI 0.409–0.674). Thin dashed line: diagonal reference line *SD* Spontaneous diuresis, *FST* Furosemide stress test, *KRT* Kidney replacement therapy

The volume of the urine output two hours following FST was also significantly associated with a successful liberation from KRT (AUC 0.871; *p* < 0.001) with a urine output of 490 ml setting the best cutoff. In contrast, the 24-h SD before was not associated with a successful liberation from KRT in the following 7 d (AUC 0.542; *p* = 0.531) (Fig. 3). If a urine output > 490 ml would be used to define FST positivity, the positive predictive value (0.961) and specificity (0.926) would increase, although at the expense of a lower sensitivity (0.690) and a lower negative predictive value (0.532) (Table 3).

**Table 3:** Furosemide stress test and successful liberation from kidney replacement therapy within 7 days after recovery of spontaneous diuresis

Parameter	All n = 98	no KRT n = 71	KRT n = 27	*p*
2-h diuresis after furosemide (ml)	500 [201; 900]	700 [400; 1000]	125 [80; 350]	** < 0.001**
*FST (cutoff ≥ 200 ml)*				
FST positive n (%)	76 (77.6)	65 (85.5)	11 (14.5)	** < 0.001**
FST negative n (%)	22 (22.4)	6 (27.3)	16 (72.7)	
*FST (cutoff ≥ 490 ml)*				
FST positive n (%)	51 (52.0)	49 (96.1)	2 (3.9)	** < 0.001**
FST negative n (%)	47 (48.0)	22 (46.8)	25 (53.2)	

Significant p-values 0.05 were highlighted in bold

Data presented as *n* (%) or median [25 th, 75 th quantile]

*FST* Furosemide stress test*, KRT* Kidney replacement therapy

### Other predictors of successful liberation from KRT

Mechanical ventilation, duration of KRT, the SOFA score, MAP, as well as serum creatinine, urea, potassium and urine osmolality at time of FST were also significantly associated with successful liberation from KRT in univariate analysis (Table 4).

**Table 4:** Other parameters and successful liberation from kidney replacement therapy within 7 days after recovery of spontaneous diuresis

Parameter	All n = 98	no KRT n = 71	KRT n = 27	*p*
Mechanical ventilation n (%)	34 (34.7)	16 (22.5)	18 (66.7)	** < 0.001**
Vasopressors n (%)	18 (18.4)	12 (16.9)	6 (22.2)	0.567
Duration of KRT (d)	3 [3; 6]	3 [3; 4]	6 [3; 11]	**0.006**
SOFA	7 [5; 9]	6 [4; 8]	9 [6; 12]	** < 0.001**
APACHE II	24 ± 6	23 ± 5	26 ± 7	0.063
MAP (mmHg)	81 [71; 92]	84 [73; 92]	72 [64; 83]	**0.028**
Creatinine (µmol/l)	118 [82; 201]	100 [71; 155]	186 [111; 282]	** < 0.001**
Urea (mmol/l)	11.1 [6.9; 16.6]	10.2 [6.0; 14.1]	14.8 [10.4; 21.0]	**0.002**
Potassium (mmol/l)	4.3 [4.0; 4.5]	4.2 ± 0.2	4.5 ± 0.3	**0.004**
PH	7.43 [7.39; 7.47]	7.43 [7.38; 7.46]	7.43 [7.40; 7.48]	0.811
Bicarbonate (mmol/l)	26.8 [24.9; 28.6]	26.8 [24.9; 28.6]	26.6 [24.8; 29.5]	0.946
Dose of furosemide (mg)	120 [100; 130]	120 [100; 131]	120 [100; 130]	0.558
24-h diuresis before FST (ml)	650 [500; 993]	700 [500; 1110]	603 [483; 923]	0.530
Urin osmolality (mosmol/kg)	402 [363; 484]	441 [387; 515]	372 [331; 392]	** < 0.001**

Significant p-values 0.05 were highlighted in bold

Data presented as *n* (%), mean ± standard deviation or median [25 th, 75 th quantile]

*APACHE II* Acute Physiology And Chronic Health Evaluation II, *FST* Furosemide stress test*, IgG* Immunoglobulin G*, KRT* Kidney replacement therapy, *MAP* Mean arterial pressure, *SOFA* Sequential organ failure assessment

After backward elimination three variables remained significant in the multivariate logistic regression model. Including these in the final model, positive FST (*p* < 0.001, OR 34.65, CI 6.58–182.40), absence of mechanical ventilation (*p* < 0.001, OR 13.15, CI 3.05–56.77) and serum urea (*p* = 0.005, OR 1.13, CI 1.04–1.23) could be identified as independent predictors for successful liberation from KRT. Even using the best cut-off from the ROC analysis for FST positivity, which was 490 ml/2 h, did not change the results of the logistical regression model. The positive FST (*p* < 0.001, OR 22.42, CI 4.41–114.04) and the absence of mechanical ventilation (*p* < 0.001, OR 8.84, CI 2.45–31.87) remained as independent parameters.

### Outcome data

There were no significant differences between FST positive and FST negative patients regarding ICU stay, ICU-, 28-day-, and 90-day-mortality and neither the need for dialysis at day 90 (Table 5).

**Table 5:** Clinical outcome depending on the result of furosemide stress test

Parameter	All n = 98	FST positive n = 76	FST negative n = 22	*p*
ICU stay (days)	10 [5; 24]	9 [5; 17]	18 [5; 31]	0.371
ICU mortality n (%)	10/98 (10.2)	7/76 (9.2)	3/22 (13.6)	0.689
28-day mortality n (%)	23/94 (24.5)	18/73 (24.7)	5/21 (23.8)	0.937
90-day mortality n (%)	35/82 (42.7)	27/61 (44.3)	8/21 (38.1)	0.622
Dialysis dependence (day 90) n (%)	4/72 (5.6)	2/53 (3.8)	2/19 (10.5)	0.567

Data presented as *n/n of available data* (%)

*FST* Furosemide stress test*, ICU* intensive care unit

### Adverse events

Hypokalemia, arrhythmias and hypotension did not differ in the period 24 h before and after furosemide administration. However, more potassium was supplemented in the 24-h period after FST, whereas the norepinephrine requirement was higher in the 24 h before. (Additional file 5).

## Discussion

In the present study, a positive FST was able to predict successful liberation from KRT during the next 7 days with a 13.1% higher absolute probability than the recovery of SD of at least 400 ml/24 h alone without any diuretic therapy. Conversely patients with negative FST had an approximately five- to six-fold-probability for the need to restart KRT. The cut-off 200 ml diuresis two hours after FST demonstrated a good prediction for successful liberation from KRT. In comparison using the cut-off 490 ml would increase the positive predictive value and specificity, but at the expense of a lower negative predictive value and sensitivity.

Mechanical ventilation, duration of KRT, the SOFA score, MAP, as well as serum creatinine, urea and potassium at time of FST were associated with early recovery of kidney function in this study, which was similar to previous findings [13]. However, there is heterogeneity of many factors regarding the prediction of kidney recovery between studies [13, 15, 18, 19]. The kinetics of eGFR [17] and creatinine clearance [16, 38] were reported as markers of successful liberation from KRT, but were not investigated in the present trial. A biomarker-centric approach to detect early renal recovery might be another opportunity. Candidates include angiotensinogen, cystatin C, hepatocyte growth factor, IL-18, KIM-1, LFABP, microRNA, NAG, NGAL, CCL14, PENK and uCHI3L1 [13, 15, 20, 23, 24, 39]. In particular, CCL14 appears to be the best predictor of stage 3 AKI persistence (AUC 0.83) [23].

After multivariate logistic regression, only positive FST and the absence of mechanical ventilation remained independent predictors for successful liberation from KRT in this study. This was similarly shown for the positive FST in a small cohort [32]. Nevertheless, in contrast to our study design, the urine output during the 24 h prior to FST was not defined in that study. This precondition was recommended in previous publications in order to enhance the robustness of FST [25].

The connection between mechanical ventilation and termination of KRT has not yet been proven [15, 18, 19]. Mechanical ventilation leads to a deterioration in kidney function due to disturbances in gas exchange, has effects on renal blood flow and perpetuates the release of inflammatory mediators [40]. This crosstalk between lung and kidney could play a role in ventilator-induced kidney injury [41]. It seems plausible that first, this pathophysiological crosstalk and second, the lack of need for mechanical ventilation, as expression of a general critical ill condition, is already associated with recovery of kidney function.

Data on serum urea and successful liberation from KRT are limited [39]. A daily urea excretion of > 1.35 mmol/kg/24 h and a urine urea concentration of > 148 mmol/l are associated with successful liberation from KRT [42], but are probably dependent from serum urea and other factors. In this issue must be taken into account that the serum urea concentration in the study population is considerably influenced by KRT.

Using the cut-off 490 ml/2 h, FST and no mechanical ventilation remained also highly significantly associated with successful liberation from KRT using the same multivariate logistic regression model. However, the odds ratios were lower than for the cut-off of 200 ml/2 h despite the higher positive predictive value and specificity in the primary analysis. Against this background, it must be critically questioned whether an increase in the cut-off to 490 ml/2 h really increases the selectivity for a successful liberation from KRT.

In this study patients with early recovery of kidney function had a significantly higher urine osmolality. An old study from 1973 showed that in the absence of renal recovery, urine osmolality remains at levels around 350 mosmol/kg, which corresponds to a free water clearance close to zero [43]. Accordingly, an increase in urine osmolality is associated with early recovery of tubular epithelial cells [43]. This relationship forms the pathophysiological basis for the FST. Provided that a certain glomerular filtration takes place, furosemide has a tubular secretion and inhibits the apical Na–K-2 Cl channel in the ascending thick part of the loop of Henle. If dysfunction of tubular epithelial cells persists, no increase in diuresis is to be expected with furosemide, since primary urine passes unprocessed through the nephron. The increase of urine output by furosemide, now indicates the start of tubular urine processing and the recovery of tubular epithelial cells. This would correspond to a higher GFR with the same amount of diuresis in advance. Since the natriuretic effect of furosemide lasts for about 2 h after intravenous administration, it is useful to measure the urine output for the same period [29, 44, 45].

The FST result was associated with short-term renal recovery, but not with the survival rate. Our data may seem in contrast to the findings of the other FST cohort [32]. There, the success criterion was classified as being alive and free of dialysis for at least 7 days. For this reason, it is not surprising that the successful group survived more often and is more free of KRT after days 28 and 90 [32].

Studies on the utility of FST in predicting severity of kidney injury compared non-progressors with patients who developed severe AKI or require KRT, rather than the FST positive with the FST negative ones as in our study [28, 30, 31].

Remarkably, the median ICU stay for the FST-positive patients was only half of that for FST negative patients. However, this difference was not significant, possibly due to the small group size. In general, the critical condition of the included intensive care patients might be more important.

All included patients had a mean APACHE II score of 31 points at admission. This would correspond to a predicted hospital mortality of 53% [46]. The lower observed mortality (28 d: 24.5%; 90 d: 42.7%) can be explained by a positive selection bias as only those patients who developed SD during KRT were investigated. In a large meta-analysis of studies that compared early versus delayed initiation of KRT, 28-d mortalities of 43–44% and 90-d mortalities of 55–56% were described [8].

Continuing unnecessary KRT should be avoided at all costs. Life-threatening complications can occur due to vascular access, extracorporeal circulation and anticoagulation. Disturbances of the acid–base and electrolyte balance are common and also dangerous. Furthermore, there is a loss of micro- and macronutrients as well as medication [12]. Difficult patient mobilization [12, 47] and enormous economic burden on the health system are additional negative effects of unnecessary treatments [48].

Possible side-effects of furosemide such as hypokalemia, arrhythmia, and hypotension did not differ comparing the period 24 h before and after the furosemide administration. However, the need for potassium supplementation was higher after FST, indicating significant renal potassium loss, so that potassium monitoring after FST should be recommended. The higher norepinephrine doses in the 24 h before FST can be explained by the more critical condition during this time.

Given this background, the FST is a simple and widely available diagnostic tool to assess the requirement for continuation of KRT. Potentially harmful effects of furosemide administration, such as volume depletion, electrolyte disturbances and ototoxicity, should be considered [43]. While furosemide administration does not appear to be problematic in patients with AKI, hypovolemia should be avoided [49].

### Strengths

In the present study, a 24-h SD of at least 400 ml without diuretics was defined as a criterion for attempting to stop KRT. The intention of the study was to demonstrate the additional diagnostic benefit of the FST. The number of cases appeared to be sufficiently powered to estimate the primary outcome giving the robust results of ROC-curves and the independent association of FST in the multivariable regression analysis. The characteristics of the population studied seem to represent well the patients in a daily practice of a medical ICU of a tertiary hospital.

### Limitations

First, it is a single center observational study with a limited number of cases. Secondly, the physicians were not blinded because blinding the fluid balance was not considered medically justifiable. Third, not all patients requiring restart of KRT met a hard KRT indication. Fourth, the risk of attrition and selection bias cannot be completely ruled out, as some patients could not be included in the study for medical reasons, missing informed consent and lack of capacities. However, an attempt was made to counteract this leaving the indication to restart KRT in the hands of intensivists who were not involved in the study.

## Conclusions

A positive FST appears to be a good predictor of early recovery of kidney function, if spontaneous urine output of at least 400 ml/d is observed under KRT, while a negative FST in the same setting often indicates the need for renewed KRT in the next 7 days.

## Supplementary Information

Additional file1

Additional file2

Additional file3

Additional file4

Additional file5

## Data Availability

No datasets were generated or analysed during the current study.

## Abbreviations

AKI: Acute kidney injury
APACHE II: Acute physiology and chronic health evaluation II
AUC: Area under curve
BMI: Body mass index
CCL14: Chemokine (C–C Motif) Ligand 14
d: Days
CI: Confidence interval
CVVHD-CiCa®: Continuous veno-venous hemodialysis with regional citrate anticoagulation
eGFR: Estimated glomerular filtration rate
FABP1: Fatty acid-binding protein 1
FST: Furosemide stress test
GFR: Glomerular filtration rate
h: Hours
ICU: Intensive care unit
IgG: Immunoglobulin G
IL-18: Interleukin 18
KDIGO: Kidney disease improving global outcomes
KIM-1: Kidney injury molecule-1
KRT: Kidney replacement therapy
MAP: Mean arterial pressure
NAG: N-acetyl-beta-D-glucosaminidase
NGAL: Neutrophil gelatinase-associated lipocalin
PENK: Proenkephalin
RCA: Regional citrate anticoagulation
ROC: Receiver operating characteristic
SOFA: Sequential organ failure assessment
uCHI3L1: Urine chitinase 3-like protein 1
