# Supplementary material for: Spontaneous diuresis in combination with furosemide stress test (SD-FST) as predictor for successful liberation from kidney replacement therapy: a prospective observational study
Source: Crit Care. 2025 May 26;29:214. doi: 10.1186/s13054-025-05452-1 (PMC12107999; doi:10.1186/s13054-025-05452-1)
Supplement: Supplementary file 2 — Additional file2 [file 13054_2025_5452_MOESM2_ESM.docx]

**Additional file 2 Indications to start initial KRT**

| **Parameter** | **All**  **n=98** | **FST positive**  **n=76** | **FST negative**  **n=22** | **p** |
| --- | --- | --- | --- | --- |
| **Reasons to start KRT:** |  |  |  |  |
| acidosis (pH < 7,25) n (%) | 44 (44.9) | 36 (47.4) | 8 (36.4) | 0.361 |
| high potassium level (> 6 mmol/l) n (%) | 13 (13.3) | 10 (13.2) | 3 (13.6) | 1.000 |
| azotemia (urea > 25 mmol/l) n (%) | 42 (43.3) | 32 (42.7) | 10 (45.5) | 0.816 |
| Oliguria/Anuria n (%) | 27 (41.5) | 18 (36.0) | 9 (60.0) | 0.098 |
| hypervolemia n (%) | 20 (20.4) | 19 (25.0) | 1 (4.5) | 0.067 |
| creatinine (µmol/l) | 226 [164; 385] | 217 [160; 366] | 259 [173; 466] | 0.219 |

Data presented as *n* (%) or median [25th, 75th quantile]

*FST* Furosemide stress test*, KRT* Kidney replacement therapy
