# Supplementary material for: Spontaneous diuresis in combination with furosemide stress test (SD-FST) as predictor for successful liberation from kidney replacement therapy: a prospective observational study
Source: Crit Care. 2025 May 26;29:214. doi: 10.1186/s13054-025-05452-1 (PMC12107999; doi:10.1186/s13054-025-05452-1)
Supplement: Supplementary file 3 — Additional file3 [file 13054_2025_5452_MOESM3_ESM.docx]

**Additional file 3 Indications to restart KRT during the first 7 days after FST**

| **Indications to restart KRT:** | **KRT**  **n=27** |
| --- | --- |
| high potassium level (> 6 mmol/l) n (%) | 1 (3.7) |
| azotemia (urea > 25 mmol/l) n (%) | 20 (74.1) |
| oliguria/anuria n (%) | 7 (26.9) |
| hypervolemia n (%) | 15 (55.6) |
| encephalopathy n (%) | 13 (48.1) |
| creatinine (µmol/l) | 288 [170; 432] |

Data presented as *n* (%) or median [25th, 75th quantile]

*FST* Furosemide stress test*, KRT* Kidney replacement therapy
