# Supplementary material for: Spontaneous diuresis in combination with furosemide stress test (SD-FST) as predictor for successful liberation from kidney replacement therapy: a prospective observational study
Source: Crit Care. 2025 May 26;29:214. doi: 10.1186/s13054-025-05452-1 (PMC12107999; doi:10.1186/s13054-025-05452-1)
Supplement: Supplementary file 4 — Additional file4 [file 13054_2025_5452_MOESM4_ESM.docx]

**Additional file 4 FST and the need to restart KRT within 7 days in different KRT modalities**

| **Parameter** | **All** | **no KRT** | **KRT** | **p** |
| --- | --- | --- | --- | --- |
| **CVVHD-CiCa^®^** | 83 | 64 | 19 |  |
| FST positive n (%) | 68 (81.9) | 58 (85.3) | 10 (14.7) | **<0.001** |
| FST negative n (%) | 15 (18.1) | 6 (40.0) | 9 (60.0) |  |
| **SLEDD** | 15 | 7 | 8 |  |
| FST positive n (%) | 8 (53.3) | 7 (87.5) | 1 (12.5) | **<0.001** |
| FST negative n (%) | 7 (46.7) | 0 (0.0) | 7 (100.0) |  |

Data presented as *n* (%)

*CVVHD-CiCa®* Continuous veno-venous hemodialysis with regional citrate anticoagulation, *FST* Furosemide stress test*, KRT* Kidney replacement therapy, *SLEDD* slow extended daily dialysis
