# Supplementary material for: Spontaneous diuresis in combination with furosemide stress test (SD-FST) as predictor for successful liberation from kidney replacement therapy: a prospective observational study
Source: Crit Care. 2025 May 26;29:214. doi: 10.1186/s13054-025-05452-1 (PMC12107999; doi:10.1186/s13054-025-05452-1)
Supplement: Supplementary file 5 — Additional file5 [file 13054_2025_5452_MOESM5_ESM.docx]

**Additional file 5 Adverse events after Furosemide administration**

| **Parameter** | **24 h before FST** | **24 h after FST** | **p** |
| --- | --- | --- | --- |
| Potassium < 3.5 mmol/l n (%) | 6/98 (6.1) | 12/98 (12.2) | 0.210^*^ |
| KCl supplemetation n (%) | 21/98 (21.4) | 34/98 (34.7) | 0.019^*^ |
| Arrhythmia n (%) | 5/98 (5.1) | 3/98 (3.1) | 0.625^*^ |
| MAP < 65 mmHg n (%) | 67/98 (68.4) | 63/98 (64.3) | 0.523^*^ |
| NA (µg/kg/min) | 0.00 [0.00, 0.09] | 0.00 [0.00, 0.01] | <0.001^#^ |

^*^McNemar test, ^#^Wilcoxon signed-rank test

Data presented as *n* (%) or median [25th, 75th quantile]

*FST* Furosemide stress test*, KCl* Potassium chloride, *MAP* mean arterial pressure, *NA* Noradrenaline
